# Abnormal Glucose Metabolism in Rheumatoid Arthritis

**DOI:** 10.1155/2017/9670434

**Published:** 2017-04-26

**Authors:** Hui Pi, Haotong Zhou, Huan Jin, Yaogui Ning, Youlian Wang

**Affiliations:** ^1^Department of Rheumatology, Jiangxi Provincial People's Hospital, Nanchang 330006, China; ^2^Department of Critical Care Medicine, The First Affiliated Hospital of Xiamen University, Xiamen 361003, China

## Abstract

The incidence of abnormal glucose metabolism in patients with rheumatoid arthritis was considerably higher than the general population. The persistent systemic inflammatory state in rheumatoid arthritis might be associated with the glucose metabolism dysfunction. In this context, insulin resistance, islet *β* cell apoptosis, inflammatory cytokines, and other aspects which were linked with abnormal glucose metabolism in rheumatoid arthritis were reviewed. This review will be helpful in understanding the abnormal glucose metabolism mechanism in patients with rheumatoid arthritis and might be conducive to finding an effective treatment.

## 1. Introduction

Rheumatoid arthritis (RA) is a systemic autoimmune disease characterized by chronic, symmetry, and destructive poly-articular synovitis. Although its pathogenesis remains unclear, it has shown that inflammation induced by abnormal immune response plays a crucial role in the development of RA. Recent studies show that RA patients with diabetes mellitus (DM) prevalence rate was about 15% to 19%, which was significantly higher than the prevalence rate of 4% to 8% of global middle-aged population DM [1, 2]. In a study, which consists of 48,718 cases of RA patients and 40,346 cases of nonrheumatic subjects, the incidence of RA patients with DM was 0.86% higher than the 0.58% in the control group which were observed, and DM risk was 1.5-fold in RA patients when compared with control group [3]. Consistently, a study described that abnormal glucose metabolism in RA patients was up to 46% after 2 years when compared with the time point of recruitment [4]. It is seen that the impaired glucose metabolism may be accelerated progress in RA patients. Here, the abnormal glucose metabolism in RA patients was reviewed.

## 2. Inflammation and Abnormal Glucose Metabolism

### 2.1. Inflammation and Insulin Resistance (IR)

Proinflammatory cytokines (TNF-*α*, IL-6, etc.) are closely associated with IR, and its possible mechanisms include the following: (1) TNF-*α* inhibits the cascade effect of insulin signaling pathway and insulin signaling pathway through stimulating insulin receptor substrate-1 (IRS-1) phosphorylation of serine which inhibited tyrosine phosphorylation [5], reduces adipocyte glucose transport protein-4 (GLUT4) expression, and inhibits the generation of adiponectin and resistances the effect of adiponectin promote insulin sensitivity [6]; (2) TNF-*α* and IL-6 can accelerate lipolysis and improve the level of free fatty acids which was an important factor in participation in the development of IR [7]; (3) IL-1*β* reduces the expression of IRS-1 leading to impaired insulin signal transduction through an extracellular signal-regulated kinase at the transcriptional level but not a posttranscriptional pathway [8]; (4) As an anti-inflammatory cytokine, low levels of IL-10 in the human exert an insulin-sensitizing effect [9].

### 2.2. Inflammation and *β* Cell Dysfunction and Apoptosis

Studies have demonstrated that the insulin secretion of islet *β* cells stimulated by glucose was inhibited when the IL-1*β*, IFN-*γ*, and TNF-*α* were added in the islet *β* cells culture system [10]. Furthermore, IL-1*β* treatment can stimulate inducible nitric oxide synthase (iNOS) expression resulting in an increased production of NO and a decreased intracellular electron transfer mitochondrial ATP synthesis. Intracellular ATP content could inhibit the secretion of insulin, leading to *β* cell dysfunction [11]. Besides, the increased Fas/FasL expression of *β* cells would lead to cell apoptosis [12]. NF-*κ*B, the mainly downstream regulator in pancreatic *β* cell stimulated by inflammatory cytokines, inhibits production of *β* cell specific proteins, such as insulin, glucose transporter 2 (GLUT-2), and insulin promoter-1 and promotes iNOS production [13]. In the meantime, it has been shown that sulforaphane exhibits a preventive role in the cytokine-induced *β* cell insulin secretory dysfunction and apoptosis through inhibiting NF-*κ*B activation and iNOS production [14].

Apoptosis mediated by endoplasmic reticulum stress has been proved to be one of the important mechanisms in pancreatic *β* cell apoptosis induced by IL-1*β*. Combination of IL-1*β* and IFN-*γ* treatment can significantly reduce the sarcoplasmic reticulum calcium pump ATP enzyme 2b protein expression and consumption endoplasmic reticulum Ca^2+^ storage, which also is raised by the BH3-only protein (only a proapoptotic protein Bcl-2 homology domain, belonging to Bcl-2 family) through inducing NO synthesis [15]. Islet *β* cells with 4-phenyl butyrate treatment significantly reduced the cell endoplasmic reticulum stress and cell apoptosis, which was induced by IL-1*β* through regulating endoplasmic reticulum Ca^2+^ concentration and c-Jun-terminal kinase (JNK) signaling pathway activation [16]. Interestingly, IL-1 receptor antagonist (IL-1ra) treatment can significantly improve blood sugar, glycated hemoglobin, proinsulin, and insulin and pancreatic *β* cell function in the diabetic mice induced by high-fat diet. Besides, it also reduced pancreatic *β* cells apoptosis, increased proliferation of *β* cells, and improved glucose-induced insulin secretion in vitro [17, 18].

The combination of IFN-*γ* and TNF-*α* treatment promoted the islet *β* cells classic cysteine-dependent apoptosis, while the administration of TNF-*α* alone did not increase islet *β* cells apoptosis. The mechanism might be due to the expression of NF-*κ*B upregulated by TNF-*α* stimulation which exhibited an antiapoptotic effect. However, the NF-*κ*B activation induced by IFN-*γ* or IL-1*β* was considered as a proapoptotic modulator [19]. Previous studies also demonstrated that combination of IFN-*γ* and TNF-*α* treatment did not increase apoptosis in the signal transducer and activator of transcription-1 (STAT1) transfected-pancreatic *β* cell, which revealed that STAT1 played an essential role in inhibiting *β* cell apoptosis resulting from IFN-*γ* and TNF-*α* treatment [20].

### 2.3. Rheumatoid Arthritis, Inflammation, and Insulin Resistance

Inflammatory cytokines, such as TNF-*α*, IFN-*γ*, IL-6, and IL-1*β*, play a crucial role in the development of RA [21], and blockage of these cytokines' activity was applied in clinical therapy [22, 23]. Biologic therapies that target specific inflammatory cytokines could improve outcomes of RA patients and reduce disability and mortality. Inhibition of TNF-*α* activity agents was firstly confirmed as the biologic drugs for RA treatment when conventional disease modified antirheumatic drugs (DMARDs) had no effect on reducing the disease activity [24]. Until now, lots of blockage of TNF-*α* activity agents were approved in the clinical use, which were divided into two categories, anti-TNF-*α* antibody and soluble TNF-*α* receptor [25]. Furthermore, other biologics were explored and licensed for the clinical use of RA [22], for instance, IL-6, a representative cytokine featuring pleiotropic activity and redundancy. However, uncontrolled persistent production of IL-6 leads to the development of rheumatoid arthritis (RA). Tocilizumab, a humanized anti-IL-6 receptor antibody, has proved its efficacy and tolerable safety either as monotherapy or in combination with DMARDs [26].

RA leads not only to the destruction of cartilage and bone but also to system damage including cardiovascular, pulmonary, and endocrine system [27, 28]. This demonstrated a relationship between inflammatory factors and insulin resistance [29–31]. Consistently, there are increased levels of serum inflammatory markers, IL-6, in patients with type 2 diabetes, which can induce beta-cell apoptosis [19, 31–33]. Our previous study also showed that the abnormal glucose metabolism was accompanied by the increased IL-6 expression [34]. Furthermore, in line with increased IL-6 expression, the apoptosis related enzyme Caspase-3 was also markedly increased in *β* cell. In addition, TNF-*α* produced by adipose tissue macrophage was widely regarded as the critical pathogenic factor in type 2 diabetes. The prevalence of RA patients with diabetes is about 15%–19%, which is significantly higher than the global incidence rate [1, 35]. The long-term systemic inflammatory status in RA patients might be the cause of islet *β* cell damage in RA patients.

## 3. RA and Abnormal Glucose Metabolism

RA patients often showed impaired *β* cell function and insulin sensitivity [36]. Dessein and Joffe found that the higher degree of inflammation (high sensitive CRP > 1.92 mg/L) was in negative correlation with the low degree of inflammation (high sensitive CRP < 1.92 mg/L) in patients with higher levels of HOMA-IR. However, there was no significant difference between HOMA-*β* levels. These data suggested that the degree of inflammation played an important effect in the progression of IR in RA patients [37]. Besides, Chung et al. reported that the prevalence of metabolic syndrome in patients with RA (42% of long-term patients, patients with early 31%) was significantly higher than the non-RA group (11%) and also found that CRP and ESR were significantly positively correlated with the homocysteine levels in patients with RA [38]. Furthermore, Chung et al. reported that when adjustment for age, sex, race, BMI, and current use of glucocorticoids (GC) was performed, the HOMA-IR levels of patients with RA were also significantly higher in patients with SLE [39]. Nevertheless, the HOMA-IR was significantly positively correlated with IL-6 (correlation coefficient *r* = 0.63) and TNF-*α* (*r* = 0.50) in patients with RA; the decreased insulin sensitivity might be due to inflammatory cytokines in RA. A study showed that 72% of early RA patients were accompanied with insulin resistance, and the HOMA-IR of RA patient group was significantly increased compared to the age- and sex-matched control group [29, 30].

RA patients without diagnosed DM frequently performed glucose tolerance test, and these results demonstrated an increased HOMA-IR index, decreased insulin sensitivity, and reduced dynamic insulin secretion index and disposition index in RA patients when compared with general population [4]. These data suggested that the early insulin secretion damaged *β* cell after glucose stimulation. On the contrary, it also demonstrated that the HOMA-*β* levels were higher in RA patients [40]. The reason is that, in order to maintained normal fasting blood glucose levels, RA patients secreted more insulin and induced an increased HOMA-*β* index. Therefore, the HOMA-*β* index increase did not mean improvement of *β* cell function but merely to compensate for the insulin sensitivity decreases. Similarly, Ferraz-Amaro et al. also detected insulin, C-peptide, and split and intact proinsulin in the RA patients and found that, in RA patients, HOMA-IR levels were significantly higher [36, 37]. Even with the exclusion of taking GC in RA patients, the results were not changed. Furthermore, in RA patients, the proportion of proinsulin was significantly higher than the control group; it might be due to proinsulin process failing to process under the systemic inflammatory state [41–44]. Taken together, this suggested that *β* cell function in RA patients had been damaged.

The above studies of RA patients with *β* cell dysfunction described a clarified relation between the systemic inflammatory state and abnormal glucose metabolism. Recently, studies had shown that IR and *β* cell function were improved after the anti-TNF therapy of RA patients, and the prevalence of DM of RA patients with biologics was significantly decreased when compared with patients with other antirheumatic drugs treatment [45–48]. These data supported the relationship between inflammation and abnormal glucose metabolism. Additionally, the use of anti-TNF agents therapy in active RA patients with IR, the fasting blood glucose, insulin levels, and IR index were improved after the anti-TNF therapy [49–53]. These findings revealed that the TNF-*α* might be the critical pathogenic cytokine in the development of IR in RA patients. By analyzing the serine^312^ phosphorylated form (p-Ser^312^) of IRS-1 and activation with phosphorylation of protein kinase (AKT) variations before and after anti-TNF therapy in RA patients, the mechanisms of its improvement in RA patients with IR might be due to p-Ser^312^IRS-1 levels decreasing and the proportion of activation with phosphorylation of AKT increasing after the anti-TNF therapy [45].

## 4. The Influence of Liver in Abnormal Glucose Metabolism in RA

In addition to articular cartilage injury, RA also leads to significant pathogenic changes in other organs such as lung, vascular tissue, liver, and muscle [54–57]. The liver is an important organ participating in glucose metabolism, including the storage, distribution, and regulation of organismic blood glucose. There are multiple pathways of glucose metabolism existing in the liver and some of them are unique to it [58]. RA can lead to a variety of liver lesions and then result in abnormal glucose metabolism. Several studies have shown that the reactive oxygen species (ROS) participate in the generation of RA [59, 60]. High level of ROS in the liver of adjuvant-induced arthritis in rats seems to be resulting from both a deficient antioxidant defense and a stimulated prooxidant system [61, 62]. It has been reported that the livers of collagen induced arthritis rats exhibited a higher oxygen consumption, and the efficiency of mitochondrial energy transduction did not decrease [63]. The activity of NADPH2-oxidase enzyme and N-demethylase and the levels of cytochrome P-450 in the liver microsomal of adjuvant arthritic rats are significantly reduced [64, 65], indicating that the capability of formation of *β*-glucuronide conjugates and oxidative metabolism of exogenous compounds and steroids are reduced. A study also demonstrated that the reduced availability of equivalents in cytosol and the lower catalytic activities of key enzymes phosphoenolpyruvate inhibited hepatic gluconeogenesis in arthritic rats [66]. The higher activities of glucokinase and the lower activities of hepatic mitochondrial pyruvate dehydrogenase lead to increasing the uptake of hepatic glucose and the rates of glycolysis in livers of arthritic rats [67]. The systemic inflammation induced by adjuvant can cause lysosomes and mitochondria irregularly shaped and result in hepatic transaminases of the plasma with higher activities and hepatocellular morphology changes [68]. In conclusion, RA can lead to liver dysfunction and affect glucose metabolism which might result in abnormal glucose metabolism.

## 5. RA Patients Using GC with Abnormal Glucose Metabolism

Glucocorticoids (GC) was a common drug in RA treatment, but its exerts a side effect on the metabolism of RA patients [69]. In the normal population of the clinical observation study, it is found that GC treatment reduced liver and peripheral insulin sensitivity and destruction of *β* cell function [70]. Furthermore, a recent study has shown that RA patients treated with oral GC are an important risk factor for DM; a 25–30% increased risk of DM occurrence was related to each additional 5 mg current oral GCs, while only the GC doses taken persistently for 6 months are closely associated with the risk of DM [71]. The administration of GC reduced fasting insulin sensitivity in the cross-sectional study of RA patients [4, 72]. Additionally, the use of GC was mainly related to IR among women, and RA patients without other metabolic risk factors treated with low dose of GC do not lead to abnormal glucose metabolism [73]. A randomized and double-blind study also found that oral glucose tolerance test inspection after a week of the glucose metabolism did not show changes from before treatment; this situation might be because the GC itself causes abnormal glucose metabolism action offset by improving glucose metabolism in patients through anti-inflammation and disease action improvement [74]. Meanwhile, a clinical study also showed that a deterioration in glucose metabolism patients after GC treatment generally had a longer duration than before treatment; this is consistent with the aforementioned that extension of inflammatory state could increase insulin sensitivity and negative effects of *β* cell function [75]. The mechanism might be due to the fact that GC could further undermine the long course of RA patients where *β* cell dysfunction was observed. Although there are so many studies on the glucose metabolism of GC in RA patients, it still needs long-term clinical trials to study the long-term use of GC in RA patients induced diabetic effects.

## 6. Conclusion

In summary, the systemic inflammation in RA patients played an important effect in the glucose metabolism, and the long-term inflammatory status could lead to *β* cell dysfunction and apoptosis and affect the liver and hepatic glucose metabolism pathway. Besides, the drug used in the RA treatment, such as GC, had a certain influence on glucose metabolism. Therefore, the abnormal glucose metabolism in the development of RA should be paid attention, and its mechanisms need to be further explored.
